# Preoperative T and N Restaging of Rectal Cancer After Neoadjuvant Chemoradiotherapy: An Accuracy Comparison Between MSCT and MRI

**DOI:** 10.3389/fonc.2021.806749

**Published:** 2022-01-21

**Authors:** Wenjuan Liu, Yuyi Li, Xue Zhang, Jia Li, Jing Sun, Han Lv, Zhenchang Wang

**Affiliations:** ^1^ Department of Radiology, Beijing Friendship Hospital, Capital Medical University, Beijing, China; ^2^ Department of Anorectal Surgery, Jining No. 1 People’s Hospital, Jining, China; ^3^ Department of Radiology, Jining No. 1 People’s Hospital, Jining, China

**Keywords:** rectal cancer, magnetic resonance imaging, multi-slice computed tomography, neoadjuvant chemoradiotherapy, restaging

## Abstract

**Background:**

It is well established that magnetic resonance imaging (MRI) is better than multi-slice computed tomography (MSCT) for the accurate diagnosis of pretreatment tumor (T) and node (N) staging of rectal cancer. However, the diagnostic value of MRI and MSCT in local restaging of rectal cancer after neoadjuvant chemoradiotherapy (NCRT) is controversial. The aim of this study is to investigate the performance of the two imaging exams in restaging of patients with rectal cancer.

**Methods:**

Patients with rectal cancer from April 2015 to April 2021 were analyzed retrospectively. The inclusion criteria are as follows: 1) diagnosis of rectal cancer through pathology; 2) NCRT had been performed; 3) all patients had undergone both MSCT and MRI examination before the surgery. Exclusion criteria are as follows: 1) incomplete clinical and imaging data; 2) previous history of pelvic surgery. Two radiologists performed T and N staging of patient images. Diagnostic accuracy, consistency analysis, and error restaging distribution of the two imaging exams for T and N restaging of rectal cancer were assessed using postoperative pathological staging as the gold standard.

**Results:**

A total of 62 patients (49 men; mean age: 59 years; age range 29–83 years) were included in the study. The diagnostic accuracy of MSCT and MRI for T restaging was 51.6% (95% CI 39.3%–63.9%) and 41.9% (95% CI 29.6%–54.2%), respectively, and no statistical difference was found between them (*p* > 0.05). The diagnostic accuracy of MSCT and MRI for N restaging was 56.5% (95% CI 44.2%–68.8%) and 53.2% (95% CI 40.8%–65.6%), respectively, and no statistical difference was found between them (*p* > 0.05). The consistency analysis showed that T restaging (κ = 0.583, *p* < 0.001) and N restaging (κ = 0.644, *p* < 0.001) were similar between MSCT and MRI. There was no significant difference in the distribution of over, accurate, or low staging in T restaging (*p* > 0.05) and N restaging (*p* > 0.05) between MSCT and MRI.

**Conclusions:**

MSCT and MRI have similarly poor performance in the diagnosis of preoperative T and N restaging of rectal cancer after NCRT. Neither of them cannot effectively stage the ypT0-1 of rectal cancer. These findings may be of clinical relevance for planning less imaging exam.

## Introduction

Rectal cancer is the third most common malignant tumor worldwide (1), and most patients are already in an advanced stage at the time of tumor detection. The clinical practice guideline for colorectal cancer recommends preoperative neoadjuvant chemoradiotherapy (NCRT) for patients with tumor (T) 3 and/or node (N) + staging resectable rectal cancer, and NCRT must be performed for patients with T4 staging or locally advanced unresectable rectal cancer (2). Different restagings lead to different treatment schemes. For rectal cancer patients with complete clinical remission after NCRT, “watch and wait” is a new treatment strategy (3). Therefore, T and N restaging using imaging examination after NCRT for rectal cancer is very important for treatment choice.

Magnetic resonance imaging (MRI) is the most accurate imaging modality for rectal cancer because it offers the advantages of superior soft-tissue contrast, multiplanar imaging, and functional assessment (4). It is well established that MRI is better than multi-slice computed tomography (MSCT) for the accurate diagnosis of pretreatment T and N staging of rectal cancer (5–7). Although the average accuracy of MRI for T staging of rectal cancer without NCRT could reach 85% (8), it is only 52% in patients with NCRT (9). Zhan et al. (10) reported that the overall accuracy of MRI for T and N restaging was 49% and 63.8%, respectively. Necrosis, edema, and inflammatory status of peritumoral tissue, residual cancer tissue, and alternative fibrous scar tissue of rectal tumors after NCRT make great challenges for accurate restaging.

Therefore, many studies focused on the comparison of the efficacy of different imaging exams and instruments after NCRT for rectal cancer (9, 11). To our knowledge, there are few studies on the comparison of rectal cancer restaging after NCRT using MSCT and MRI. MSCT is mainly used for the staging of patients with advanced rectal cancer, especially those with other organ metastases; therefore, pelvic MSCT is always performed in patients with rectal cancer clinically. The differences in the accuracy comparison between MRI and MSCT for T and N restaging of rectal cancer after NCRT are still controversial (12).

At present, clinicians experience difficulties in selecting appropriate imaging exams in the clinical setting of restaging in rectal cancer with NCRT. They always ordered both MSCT and MRI. To determine which imaging method had better performance, this paper evaluated the diagnostic accuracy between MRI and MSCT of rectal cancer restaging after NCRT.

## Materials and Methods

### Patient Selection

Clinical data from patients with rectal cancer who were hospitalized in the general surgery department at the Beijing Friendship Hospital Affiliated to Capital Medical University between April 2015 and April 2021 were analyzed retrospectively. The following inclusion criteria were considered: 1) diagnosis of rectal cancer by pathology; 2) NCRT had been performed; 3) all patients had undergone both MSCT and MRI examination before the surgery. Exclusion criteria were as follows: 1) incomplete clinical and imaging data; 2) previous history of pelvic surgery. Age, sex, the distance of neoplasia from the anal verge, and the time interval between imaging exams and surgery were considered. All data were retrospectively collected into a dedicated database.

### Treatment

All patients in this study underwent NCRT before the surgery (13). The American Varian Clinac iX linear accelerator was used in conventional fractionated radiotherapy. The irradiation experienced by each patient was five fields, and the total dose was 5,000 cGy. The number of irradiation events was 25, and radiotherapy was performed 5 days a week for 5 weeks. Simultaneously, capecitabine tablets (Shanghai Roche Pharmaceuticals Ltd., H20073024) 1,650 mg/(m^2^·day) were given orally in the morning and evening. Patients were treated continuously for 14 days, followed by a rest period of 7 days, which together comprised one treatment cycle; all patients underwent a total of two cycles at least.

### Imaging Techniques

Before operation and after the NCRT, all patients with rectal cancer were examined by MSCT and MRI.

MSCT examinations were performed using an MSCT scanner (Lightspeed; GE Medical Systems, USA) with a 64-row detector. Scanning parameters were as follows: tube voltage 120 kV, tube current 125–300 mA, collimation slice thickness 0.5–0.75 mm, pitch 0.6–1.25, reconstruction slice thickness 3.5 mm, reconstruction interval 3–5 mm, multiplanar 3D volume rendering reconstruction slice thickness 0.5–1.0 mm, interval 0.3–0.5 mm. All patients were administered with an intravenous contrast medium (2 ml/kg; flow rate 3 ml/s; Omnipaque 320) and underwent MSCT imaging of the abdomen and pelvis—from the top of the diaphragm to the lower margin of the pubic symphysis. The arterial and venous images were collected at 25 and 60 s. Axial images were reconstructed in the coronal and sagittal planes for interactive multiplanar image viewing on a workstation.

On a separate day, MRI examinations were performed with a 3.0T unit (Discovery MR750; GE Medical Systems, USA) using a 16-channel phased array body surface coil. The scan covered the entire pelvis. In accordance with the standards for clinical routine imaging examination, the scanning sequence included T2-weighted imaging (T2WI), high-resolution small-field FSE-T2WI, diffusion-weighted imaging (DWI), and T1WI-vibe dynamic enhancement. Detailed MRI protocol is reported in **Table 1**. The b-value of DWI was set to 50 and 800 s/mm^2^; the system automatically generated the ADC diagram. Dynamic enhanced sagittal T1WI was performed last. Gadolinium-Diethylenetriaminepentaacetic acid contrast agent was injected into the vein at the back of the hand in a dose of 0.1 ml/kg, and the injection rate was 2.5 ml/s. The bladder of each patient was moderately filled, and the intestines were unprepared before MRI scanning.

**Table 1:** MRI protocol for rectal cancer.

	Sagittal T2WI	Axis high-resolution T2WI	Coronal high-resolution T2WI	Axial DWI	Contrast-enhanced sagittal T1WI
TR (ms)/TE (ms)	6,930/117	3,000/87	3,000/107	4,300/58	3.14/1.17
FOV (mm)	270 × 270	240 × 240	240 × 240	360 × 360	350 × 350
Number of signal average	2	2	2	2	2
Slice thickness (mm)	3.5	3	3	5	2.5

MRI, magnetic resonance imaging; T2WI, T2-weighted imaging; DWI, diffusion-weighted imaging; T1WI, T1 weighted image; TR, time of repetition; TE, time of echo; FOV, field of view.

### Image Analysis

All images were analyzed and reviewed at a workstation. T and N restaging of MSCT and MRI images was analyzed separately by two radiologists (XZ and WL) with different experiences (8 years and 13 years, respectively) for the interpretation of pelvic CT and MRI studies. In case of disagreement, consensus was reached after consultation. Each radiologist was aware that patients had been referred for rectal cancer restaging but was unaware of the final operative and histopathologic results. Overall, T and N restaging criteria were based on the tumor-node-metastasis (TNM) standard developed by the American Joint Committee on Cancer (AJCC) (14).

T staging criteria of MSCT were as follows (15): absence of intestinal wall thickening was defined as T0; intraluminal projection of a lesion without any visible distortion of the bowel wall layers was classified as T1; patients with asymmetrical thickening projecting intraluminally, smooth preservation of muscle coat, and clear adjacent perirectal fat were classed as T2; smooth or nodular extension of a discrete mass and disruption of the muscle coat with extension into perirectal fat were classed as T3; patients with nodular penetration through the peritonealized area of the muscle coat and with tumor penetration into adjacent organs were recorded as T4.

T staging criteria of MRI were as follows: absence of intestinal wall thickening was defined as T0; tumors that appeared to be confined to the submucosa were classified as T1; abnormal tumor signals indicating tumor extension beyond the submucosa and invasion into the muscular layer, including tumors in which the edge of the muscular layer of the intestinal wall was smooth and had a clear relationship with perirectal fat, were classed as T2; the staging criteria for T3 and T4 were the same as those for MSCT.

For MSCT and MRI N staging, lymph nodes with a transverse diameter >5 mm, fuzzy boundary, irregular shape, and non-homogeneous enhancement were considered positive for metastases. An absence of enlarged lymph nodes was recorded as N0. Patients with ≤3 enlarged lymph nodes in the mesorectal region were classed as N1. Patients with ≥4 visibly enlarged lymph nodes in the mesorectal region were classed as N2.

### Histopathology

Surgical specimens were evaluated by the same team of pathologists, and findings were reported according to the AJCC post-NCRT tumor-node-metastasis (ypTNM) classification (14). The histopathologic T stage after NCRT (ypT) was based on the deepest tissue invaded by residual tumor cells in surgical specimens.

### Statistics

T and N restaging of MSCT and MRI after NCRT was compared with corresponding histopathology. The accuracy of each staging was expressed as a percentage: accuracy (%) = the number of cases with correct stage/the number of cases with pathological gold standard. The total accuracy and 95% confidence interval (CI) of MSCT and MRI were calculated. The comparison between MSCT and MRI was analyzed with paired chi-square test (McNemar test). The κ consistency test was used to evaluate whether the restaging effects of MSCT and MRI were consistent. We used chi-square test (2×C) to analyze the differences among the data of overstaging, accurate staging, and low staging. Statistical significance was set at *p* ≤ 0.05. Statistical analyses were carried out with SPSS software (SPSS 26.0; IBM Corp., Armonk, NY, USA).

## Results

### Study Population

**Figure 1:**
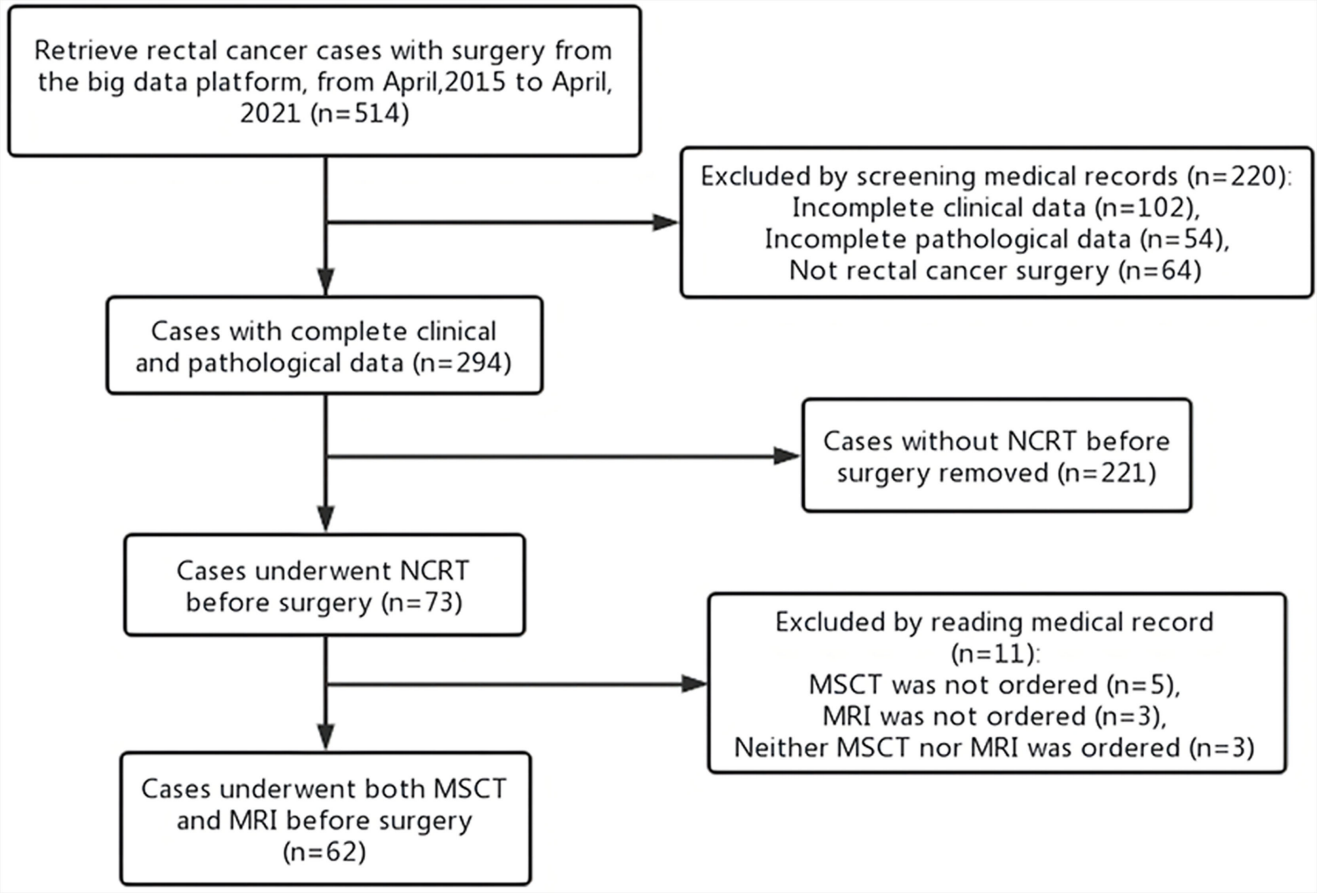
Participant inclusion flowchart. NCRT, neoadjuvant chemoradiotherapy; MSCT, multi-slice computed tomography; MRI, magnetic resonance imaging.

From an initial population of 514 patients with rectal cancer, 62 patients (49 men; mean age: 59 years; age range 29–83 years) met the inclusion and exclusion criteria and formed the final study group, as shown in the flowchart below (**Figure 1**). Detailed demographics, tumor, and imaging exam time characteristics are summarized in **Table 2**.

**Table 2:** Demographics, tumor, and imaging exam time characteristics in the 62 study patients.

Variable	
Age, median (range, years)	59 (29–83)
Sex, *n* (%)	
Male	49 (79.0)
Female	13 (21.0)
Tumor location, *n* (%)*	
Lower rectum	34 (54.8)
Middle rectum	26 (41.9)
Upper rectum	2 (3.3)
Tumor distance from anal verge, median (range, cm)	5.8 (2.0–15.0)
Time interval between MSCT and MRI (range, day)	1 (0–4)
Time interval between MSCT and surgery (range, day)	6 (3–11)
Time interval between MRI and surgery (range, day)	6 (2–11)

MSCT, multi-slice computed tomography; MRI, magnetic resonance imaging.

### T Stage

The distribution of ypT, MSCT, and MRI staging is summarized in **Table 3**.

**Table 3:** Diagnostic results of MSCT, MRI, and pathological examination for T restaging of rectal cancer after NCRT.

Pathological staging	Cases	T0		T1		T2		T3		T4		Accuracy (%)	
		MSCT	MRI	MSCT	MRI	MSCT	MRI	MSCT	MRI	MSCT	MRI	MSCT	MRI
ypT0	3	0	0	0	0	1	1	1	2	1	0	0	0
ypT1	8	1	1	0	0	3	3	4	4	0	0	0	0
ypT2	20	0	0	0	0	6	4	13	15	1	1	30	20
ypT3	28	0	0	0	0	3	5	24	20	1	3	85.7	75
ypT4	3	0	0	0	0	0	1	1	0	2	2	66.7	66.7
Total	62	1	1	0	0	13	14	43	41	5	6	51.6	41.9

MSCT, multi-slice computed tomography; MRI, magnetic resonance imaging; NCRT, neoadjuvant chemoradiotherapy; T, tumor; yp, pathological staging after neoadjuvant chemoradiotherapy.

On the basis of pathological staging, 3 patients (4.8%) were diagnosed ypT0, 8 patients (13.0%) were diagnosed with ypT1, 20 patients (32.2%) were diagnosed with ypT2, 28 patients (45.2%) were diagnosed with ypT3, and 3 (4.8%) had a ypT4 rectal cancer. Neither MSCT nor MRI could accurately diagnose ypT0-1 staging. There was a fair agreement between MSCT and MRI findings for T restaging of rectal cancer after NCRT (κ = 0.583, *p* < 0.001). The T restaging accuracy of MSCT and MRI was 51.6% (95% CI 39.3%–63.9%) and 41.9% (95% CI 29. 6%–54.2%), respectively, and there was no significant difference between the two examination methods (*p* > 0.05) (**Table 4**).

**Table 4:** Comparison of accuracy between MSCT and MRI for T/N restaging of rectal cancer after NCRT.

		MSCT		
		True (T/N)	False (T/N)	
MRI	True (T/N)	22/28	3/4	25/32
	False (T/N)	10/7	27/23	37/30
		32/35	30/27	62/62

MSCT, multi-slice computed tomography; MRI, magnetic resonance imaging; T, tumor; N, node; NCRT, neoadjuvant chemoradiotherapy.

### N Stage

The distribution of ypN, MSCT, and MRI staging is summarized in **Table 5**. On the basis of pathological staging, 46 patients (74.2%) were diagnosed ypN0, 9 patients (14.5%) were diagnosed with ypN1, and 7 (11.3%) had a ypN2 rectal cancer. There was a fair agreement between MSCT and MRI findings for N restaging of rectal cancer after NCRT (κ = 0.644, *p* < 0.001). The N restaging accuracy of MSCT and MRI was 56.5% (95% CI 44.2%–68.8%) and 53.2% (95% CI 40.8%–65.6%), respectively, and there was no significant difference between the two examination methods (*p* > 0.05) (**Table 4**).

**Table 5:** Diagnostic results of MSCT, MRI, and pathological examination for N restaging of rectal cancer after NCRT.

Pathological staging	Cases	N0		N1		N2		Accuracy (%)	
		MSCT	MRI	MSCT	MRI	MSCT	MRI	MSCT	MRI
ypN0	46	32	29	8	10	6	7	51.6	63.0
ypN1	9	5	4	0	1	4	4	0	11.1
ypN2	7	4	3	0	1	3	3	42.9	42.9
Total	62	41	36	8	12	13	14	56.5	53.2

MSCT, multi-slice computed tomography; MRI, magnetic resonance imaging; N, node; NCRT, neoadjuvant chemoradiotherapy; yp, pathological staging after neoadjuvant chemoradiotherapy.

### Error Restaging Distribution

**Figure 2:**
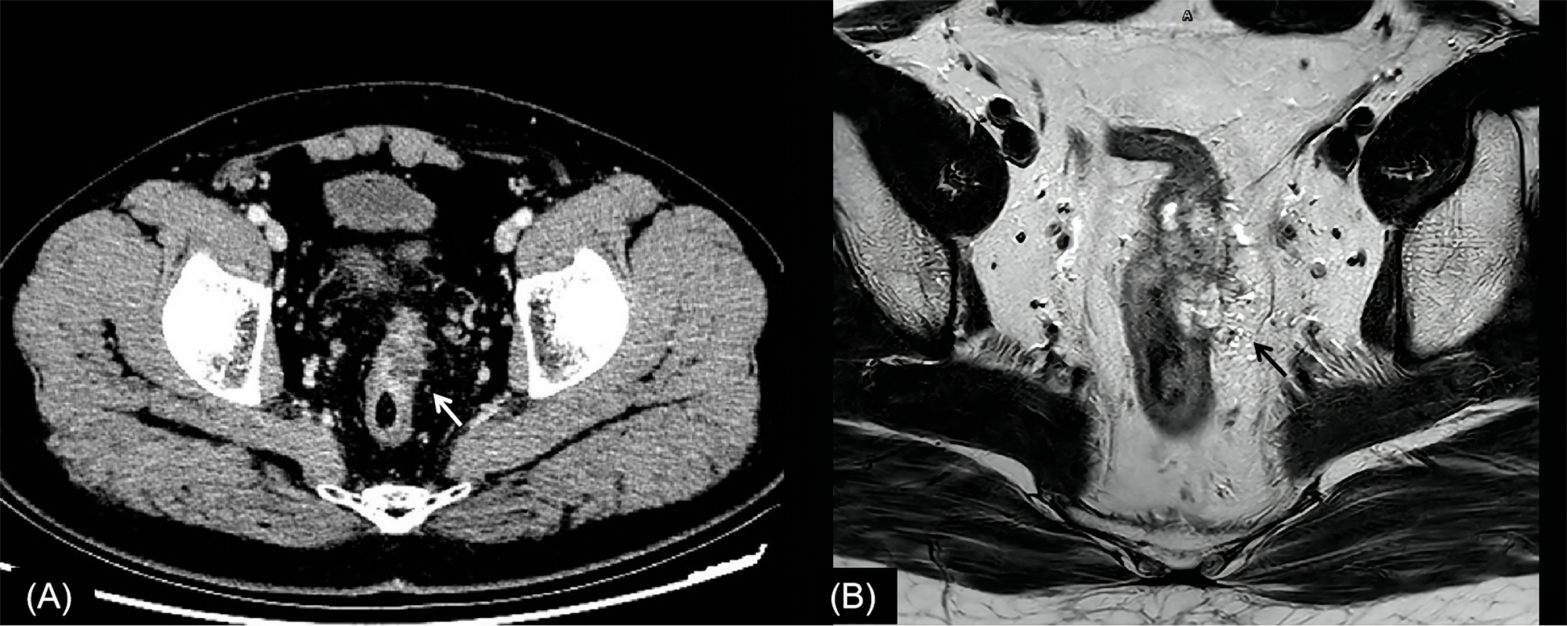
Rectal cancer with ypT0N0. **(A)** Axial MSCT depicts a heterogeneous enhancing tumor penetrating the peritoneal reflection (arrow). Over restaged as T4. **(B)** MRI transverse high-resolution T2WI of rectum shows left mesorectal fascia involvement (arrow). Over restaged as T3N0. yp, pathological staging after neoadjuvant chemoradiotherapy; T, tumor; N, node; MSCT, multi-slice computed tomography; MRI, magnetic resonance imaging; T2WI, T2-weighted imaging.

Both MSCT and MRI demonstrated over, accurate, and low staging for T and N of rectal cancer after NCRT, respectively (**Figures 2** **–** **6**). For T restaging, 24 patients (38.7%) were overstaging and 6 patients (9.7%) were low staging with MSCT; 29 patients (46.8%) were overstaging and 7 patients (11.3%) were low staging with MRI. For N restaging, 18 patients (29.0%) were overstaging and 9 patients (14.5%) were low staging with MSCT; 22 patients (35.5%) were overstaging and 7 patients (11.3%) were low staging with MRI. There were no significant differences in the distribution of overstaging, accurate staging, or low staging in T and N restaging between MSCT and MRI (*p* > 0.05) (**Table 6**).

**Figure 3:**
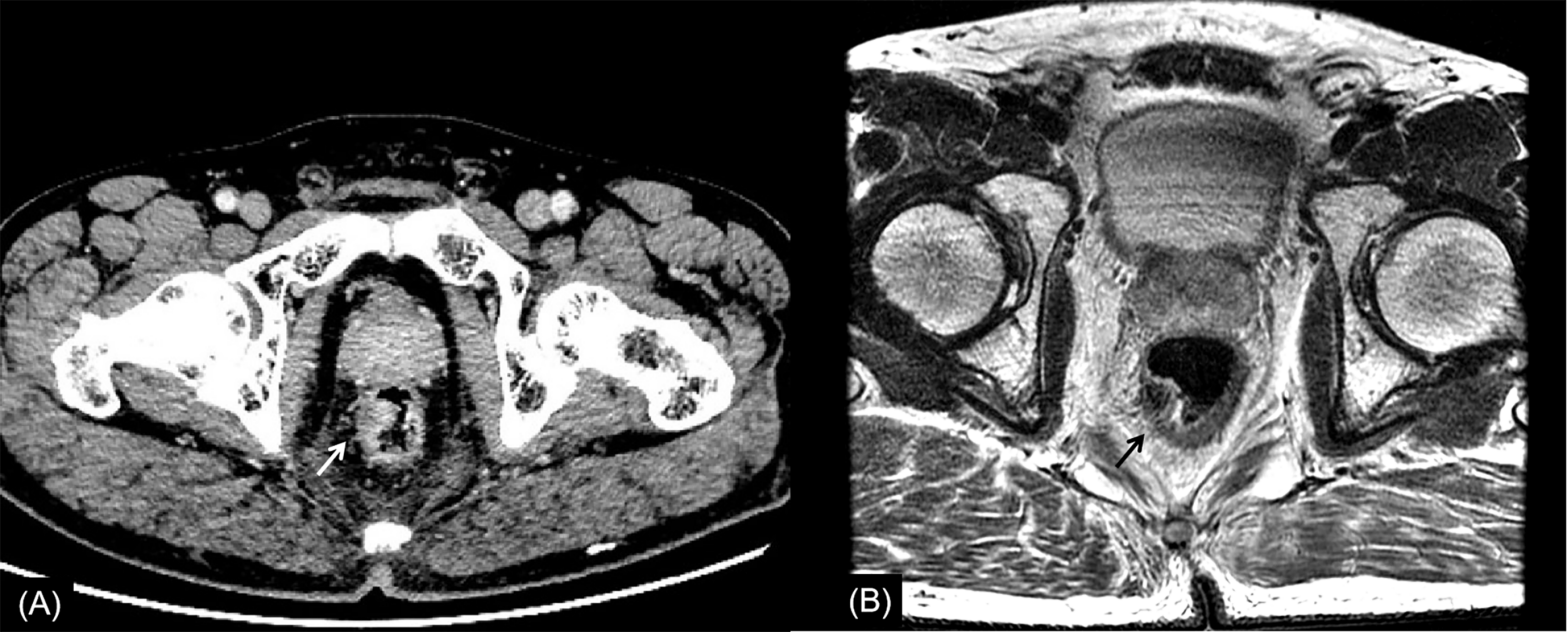
Rectal cancer with ypT1N0. **(A)** Axial MSCT depicts a mass protruding into the enteric cavity (arrow) and penetrating the muscular layer. Over restaged as T2. **(B)** MRI transverse high-resolution T2WI of rectum shows a hypointense mass involving muscular layer (arrow). Over restaged as T2. yp: pathological staging after neoadjuvant chemoradiotherapy; T, tumor; N, node; MSCT, multi-slice computed tomography; MRI, magnetic resonance imaging.

**Figure 4:**
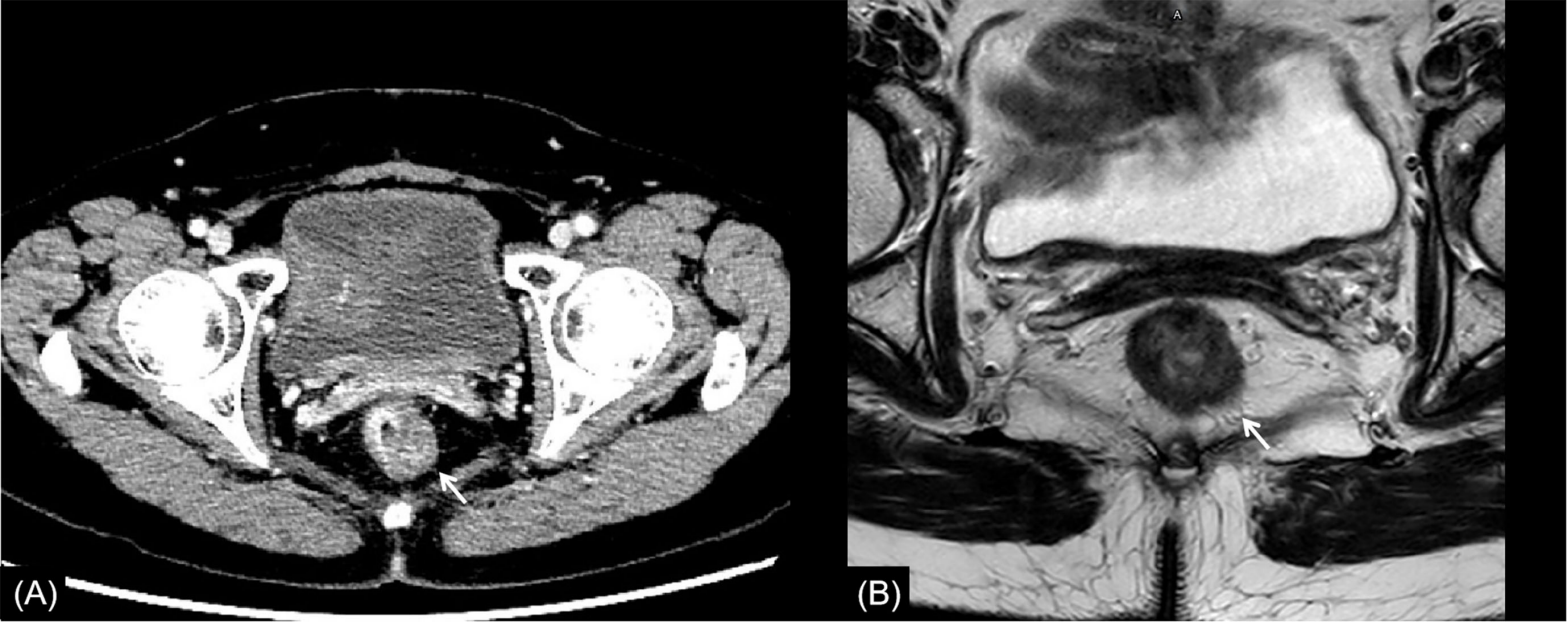
Rectal cancer with ypT2N0. **(A)** Axial MSCT depicts a heterogeneous enhancing rectal tumor (arrow) with a mild rough outer wall. Over restaged as T3. **(B)** MRI transverse high-resolution T2WI shows a heterogeneous signal mass involving left rear mesorectal fascia (arrow). Over restaged as T3. yp: pathological staging after neoadjuvant chemoradiotherapy; T, tumor; N, node; MSCT, multi-slice computed tomography; MRI, magnetic resonance imaging.

**Figure 5:**
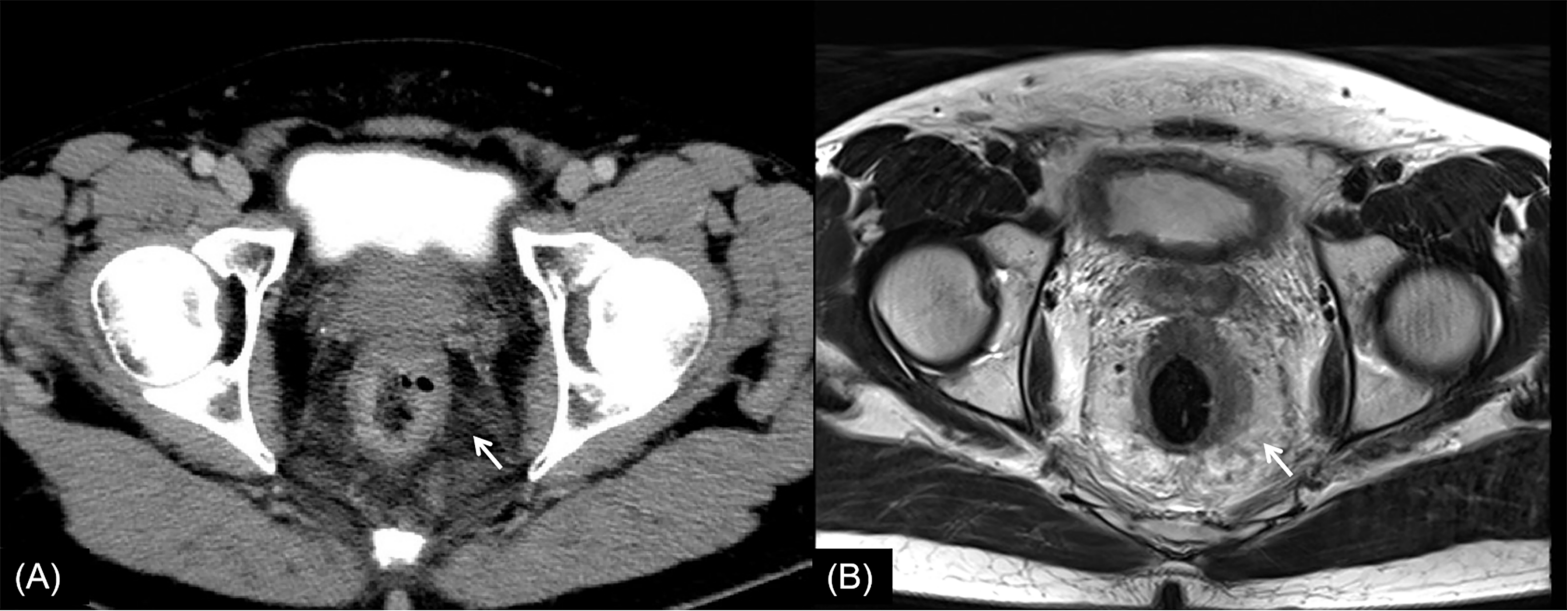
Rectal cancer with ypT3N2. **(A)** Axial MSCT depicts left rectal wall thickening and mesorectal edema (arrow). Accurately restaged as T3. **(B)** MRI transverse high-resolution T2WI shows thickened left rectal wall involving mesorectal fascia (arrow). Accurately restaged as T3. No enlarged lymph nodes were found in MSCT images. Low restaged as N0 with MSCT and MRI. yp, pathological staging after neoadjuvant chemoradiotherapy; T, tumor; N, node; MSCT, multi-slice computed tomography; MRI, magnetic resonance imaging; T2WI, T2-weighted imaging.

**Figure 6:**
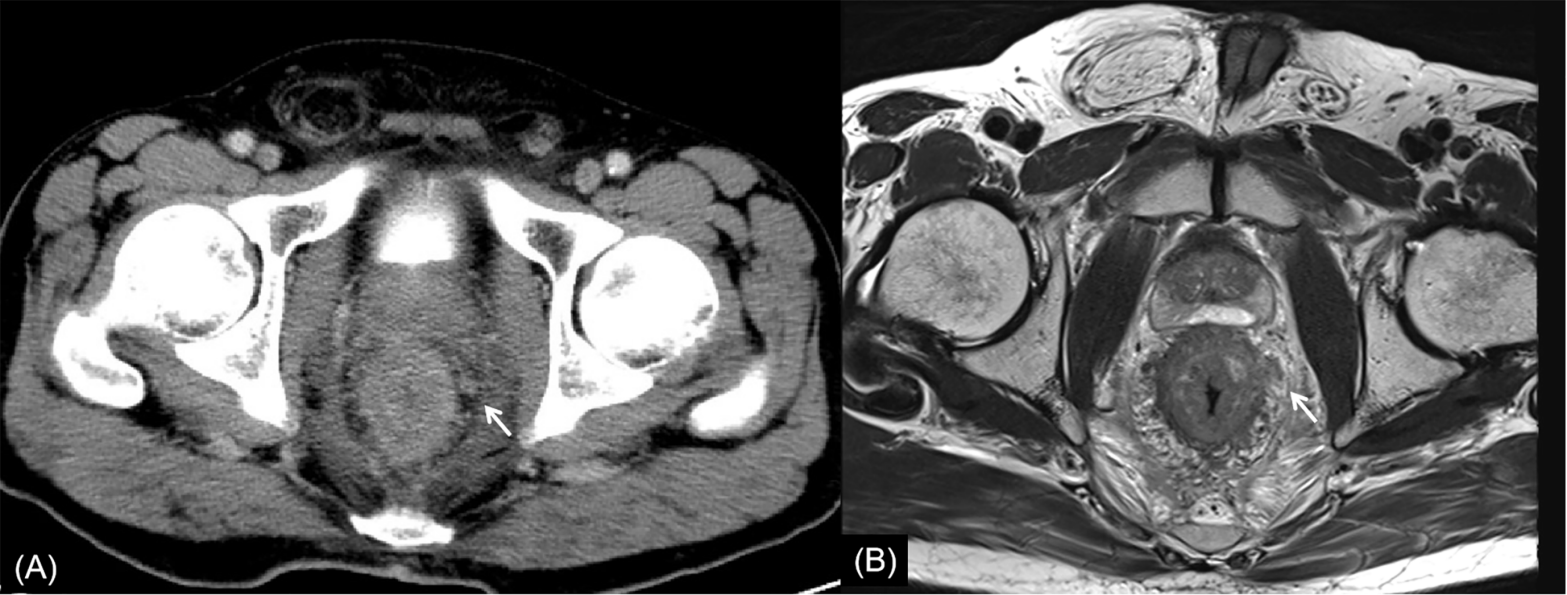
Rectal cancer with ypT4N2. **(A)** Axial MSCT depicts whole rectal wall thickening with uneven density of adjacent mesorectum. Low restaged as T3. **(B)** MRI transverse high-resolution T2WI shows thickened whole rectal wall with extensive edema and fibrosis of mesorectum. Low restaged as T3. Enlarged lymph node (arrow) was found. Accurately restaged as N2 with MSCT and MRI. yp: pathological staging after neoadjuvant chemoradiotherapy; T, tumor; N, node; MSCT, multi-slice computed tomography; MRI, magnetic resonance imaging.

**Table 6:** MSCT and MRI findings in T and N restaging of rectal cancer after NCRT [n (%)].

Methods of examination	T restaging (n = 62)			N restaging (n = 62)		
	Overstaging	Accurate staging	Low staging	Overstaging	Accurate staging	Low staging
MSCT	24 (38.7)	32 (51.6)	6 (9.7)	18 (29.0)	35 (56.5)	9 (14.5)
MRI	29 (46.8)	26 (41.9)	7 (11.3)	22 (35.5)	33 (53.2)	7 (11.3)
χ²	1.169			0.709		
*P*	0.557			0.702		

MSCT, multi-slice computed tomography; MRI, magnetic resonance imaging; T, tumor; N, node; NCRT, neoadjuvant chemoradiotherapy.

## Discussion

This study compared the accuracy of T and N restaging between MSCT and MRI in patients with rectal cancer after NCRT aimed at assessing the diagnostic value of the two imaging exams. Although the accuracy of T3 restaging could reach 85.7% (MSCT) and 75% (MRI), the overall restaging accuracy was poor and the most frequent inaccuracy was overstaging. The study also found that MSCT and MRI could not correctly restage ypT0 and ypT1. It was reported that patients with clinical complete remission would not be operated and observed closely (16). Due to the poor accuracy of restaging in preoperative imaging exams, no matter what the clinical staging after NCRT, radical surgery was still actively carried out in the clinic. The performances of MSCT and MRI for the restaging were similar in the study, so clinicians might choose one imaging exam instead of both according to clinical needs.

MRI was the main imaging modality used for local staging of rectal cancer; MSCT was mainly used for distant metastasis staging (17). Both MRI and MSCT were equally important. Faletti et al. (18) reported that the accuracy of MRI for T and N staging of rectal cancer pretreatment can reach 90.4% and 76.9%, respectively. However, radiotherapy and chemotherapy can lead to necrosis and regression of rectal tumors, as well as fibrosis, necrosis, and other pathological reactions of connective tissue. This makes it difficult to distinguish between tumor tissue, fibrous scar tissue, and normal intestinal wall tissue, and this leads to reduced accuracy of T and N staging.

In this study, the main reason for incorrect restaging was overstaging of T0-2, and there was no significant difference between the two methods of examination. Interestingly, similar conclusions have been drawn by Pomerri et al. (12). But they reported that the accuracy of ypT staging was low, whatever the imaging technique used (37% by CT, 34% by MRI, and 27% by endorectal ultrasound). Different from a previous study, our study did the consistency analysis of MSCT and MRI for restaging and the statistical analysis of the differences among over, accurate, and low staging. The accuracies of CT and MRI in their study were lower than those in our study, which might be due to the limitation of machine performance in that period. One potential reason for overstaging of T0-2 in MSCT was that the density of scar tissue after NCRT was uneven, and the outer edge of the intestinal wall was not smooth (**Figures 2**, **4**). The reason for overstaging of T0-2 in MRI might be due to inflammation and edema within the fatty tissue that surrounds the tumor after NCRT. This fatty tissue appeared as a mild hyperintensity on T2WI images (**Figures 2**, **4**). The manifestations were considered as T3 staging caused by penetration into the muscle layer and infiltration into the fat layer around the intestinal wall. The ypT1 patient in **Figure 3** was restaged as T2 by MSCT and MRI. **Figure 3** shows the local rectal muscle layer and an abnormal structure of equal density/signal that was pathologically confirmed as the fibrotic component after NCRT. In some cases, the tumor components of the mesorectum were confused with edema and fibrosis after NCRT, which confused T3 and T4 (**Figure 4**).

For N restaging, the accuracy of MSCT and MRI was only 56.5% and 53.2%, respectively, and there was no significant difference between them (*p* > 0.05). Previous studies reported that the accuracy of MRI and CT in N restaging of rectal cancer after NCRT was 55% and 62%, respectively (12, 19). A study showed that neither CT density or size of lymph nodes could accurately distinguish metastatic lymph nodes from reactive proliferative lymph nodes (12). In the patient depicted in **Figure 6**, no enlarged lymph nodes were found on MSCT or MRI, but multiple metastatic lymph nodes were found in postoperative pathology testing. So, the accurate judgment of pathological restaging according to imaging exam is still a difficult problem in rectal cancer after NCRT at present from our study, no matter the T or N.

The appropriate choice of therapeutic regime after NCRT highly depends on the accuracy of local T and N restaging. The current routine imaging exams are not accurate enough, since some studies believe that MRI can provide some value in additional features such as the circumferential resection margin and extramural vascular invasion (4, 20). Therefore, radical resection is often performed in the clinic. In recent years, an increasing number of clinical studies have been conducted to evaluate the efficacy of NCRT in rectal cancer, and most studies suggest that radiomics are of high value for the evaluation of tumor regression grading and pathological complete remission (21–24). Therefore, radiomics may be one of the most promising development directions for solving this problem in the future.

Our study had some limitations. First, our series included a small number of ypT0 and ypT4 lesions. Therefore, it is difficult to determine whether our observations regarding the performance of imaging could be applied to patients with ypT0 and ypT4. Second, while this study is a retrospective case analysis, we only studied the conventional sequence of MRI and did not study the new sequence or functional imaging. To confirm our findings, future studies should include prospective, large-sample, multicenter, randomized controlled methods. Finally, this study only focused on the restaging after NCRT. In order to clarify the research goal, we did not perform in conjunction with the pretreatment test. This is another subject we want to study in the next step.

In conclusion, the diagnostic accuracies of MSCT and MRI for T and N restaging of rectal cancer after NCRT were poor and had similar performances mainly due to the overstaging of ypT0-2. Neither of the two imaging exams could effectively predict ypT0-1 staging of rectal cancer after NCRT. In general, abdominal pelvic MSCT was always ordered in the restaging clinical setting before surgery because it considered distant metastasis restaging and local partial restaging in one examination. Therefore, to save medical resources, clinicians could choose one imaging exam according to their needs rather than both.

## Data Availability Statement

The original contributions presented in the study are included in the article/supplementary material. Further inquiries can be directed to the corresponding authors.

## Ethics Statement

The studies involving human participants were reviewed and approved by Beijing Friendship Hospital, Capital Medical University. Written informed consent for participation was not required for this study in accordance with the national legislation and the institutional requirements.

## Author Contributions

The authors confirm contribution to the paper as follows: study conception and design: WL and ZW. Data collection: YL. Analysis and interpretation of results: XZ, JL, and JS. Draft article preparation: WL and HL. All authors reviewed the results and approved the final version of the article.

## Funding

This work was supported by the Beijing Friendship Hospital, Capital Medical University (yyqdktbh2020-9); Beijing Scholars Program, (2015)160; and Beijing Hospitals Authority Clinical Medicine Development of Special Funding Support (ZYLX202101).

## Conflict of Interest

The authors declare that the research was conducted in the absence of any commercial or financial relationships that could be construed as a potential conflict of interest.

## Publisher’s Note

All claims expressed in this article are solely those of the authors and do not necessarily represent those of their affiliated organizations, or those of the publisher, the editors and the reviewers. Any product that may be evaluated in this article, or claim that may be made by its manufacturer, is not guaranteed or endorsed by the publisher.
